# Vanadium Ferrocyanides as a Highly Stable Cathode for Lithium-Ion Batteries

**DOI:** 10.3390/molecules28020461

**Published:** 2023-01-04

**Authors:** Thang Phan Nguyen, Il Tae Kim

**Affiliations:** Department of Chemical and Biological Engineering, Gachon University, Seongnam-si 13120, Republic of Korea

**Keywords:** vanadium ferrocyanides, lithium-ion batteries, high voltage, Prussian blue, Prussian green

## Abstract

Owing to their high redox potential and availability of numerous diffusion channels in metal–organic frameworks, Prussian blue analogs (PBAs) are attractive for metal ion storage applications. Recently, vanadium ferrocyanides (VFCN) have received a great deal of attention for application in sodium-ion batteries, as they demonstrate a stable capacity with high redox potential of ~3.3 V vs. Na/Na^+^. Nevertheless, there have been no reports on the application of VFCN in lithium-ion batteries (LIBs). In this work, a facile synthesis of VFCN was performed using a simple solvothermal method under ambient air conditions through the redox reaction of VCl_3_ with K_3_[Fe(CN)_6_]. VFCN exhibited a high redox potential of ~3.7 V vs. Li/Li^+^ and a reversible capacity of ~50 mAh g^–1^. The differential capacity plots revealed changes in the electrochemical properties of VFCN after 50 cycles, in which the low spin of Fe ions was partially converted to high spin. Ex situ X-ray diffraction measurements confirmed the unchanged VFCN structure during cycling. This demonstrated the high structural stability of VFCN. The low cost of precursors, simplicity of the process, high stability, and reversibility of VFCN suggest that it can be a candidate for large-scale production of cathode materials for LIBs.

## 1. Introduction

The development of cathode materials with high capacity, high-voltage flatform, and high stability in metal-ion batteries is always a challenge to scientists [1,2,3,4]. The traditionally used cathode materials (NCM, LFP) in lithium-ion batteries (LIBs) have some limitations, such as the complexity of fabrication, high cost of precursors (Ni, Co), and toxicity of the fabrication or recycling process [5,6,7,8,9,10]. The NCM cathode is not ready for use after single-step fabrication and should be combined with carbon derivatives such as carbon nanotubes or graphene. Therefore, the complex processing from raw materials to the final active materials would increase the product cost [11,12,13]. Thus, the development of sustainable materials that meet cathode requirements is essential. In the last decade, Prussian blue analogs (PBAs) have received great attention owing to their metal–organic framework structure, which allows facile diffusion channels for cations (Li^+^/Na^+^/K^+^) [14,15,16,17,18]. PBAs consist of Fe ions with other metal ions (such as Na, K, Fe, Cr, Cu, and Mn) with the (CN)^−^ groups as the linkers, creating cubic or monoclinic structures, and as cages, which can capture the metal ions [19,20]. The synthesis of PBA via the solvothermal method is simple and can easily be modified by changing the metal sources [15,21,22]. Therefore, much research on PBAs for alkaline-ion-storage applications has been simulated and/or experimented. Alkaline metals consisting of Prussian blue, such as sodium/potassium ferrocyanide or their derivatives with other transition metals (Mn, Cr, V, and Fe), demonstrated high capacities for alkaline ion batteries [23,24,25,26]. For example, Qian et al. used a Na_4_Fe(CN)_6_/C composite that exhibited a high redox capacity of ~90 mAh g^−1^ in sodium-ion batteries (SIBs) [27]. Baster et al. employed sodium chromium hexacyanoferrate in SIBs and achieved a capacity of approximately 64 mAh g^−1^ [28]. Paulitsch et al. used the electrodeposition method to form a Na_2_VO_x_[Fe(CN)_6_] film as a cathode in aqueous SIBs, which exhibited a high specific capacity of ~80 mAh g^−1^ [29]. Lim et al. investigated Prussian white Na_2_Fe[Fe(CN)_6_], which exhibited a capacity of 169 mAh g^−1^, a value close to the theoretical capacity of ~171 mAh g^−1^ [30]. However, the unstable voltage flatform and fast degradation of these cathodes limit their practical application. Recently, the use of vanadium in PBA compounds has shown a highly improved voltage flatform and stability of the cathode materials [31,32]. Baster et al. fabricated sodium–vanadium hexacyanoferrate (NaVHCF) by the coprecipitation method using VCl_2_ with the mixture of Na_4_Fe(CN)_6_/NaCl in an inert atmosphere [31]. Pan et al. prepared NaVHCFs from a VOC_2_O_4_ precursor [32]. All NaVHCF materials possess high redox potentials, leading to an improvement in the stability of the cathode materials for SIBs. However, PBAs are rarely employed in LIBs. Based on this scenario, in this study, we demonstrate a facile synthesis of vanadium ferrocyanides (VFCN) by a one-step solvothermal method using a redox reaction between VCl_3_ and K_3_Fe(CN)_6_. These materials, when employed as cathode materials in LIBs, exhibited high stability. Kinetic analysis, in situ differential capacity plots, and ex situ X-ray diffraction (XRD) measurements were performed to further investigate the behavior of the VFCN cathode in LIBs.

## 2. Results and Discussion

For the synthesis of VFCN, VCl_3_ was premixed with ethanol; thus, the combination resulted in VCl_2_OC_2_H_5_ (VCl_3_ + xC_2_H_5_ → VCl_3−x_(OC_2_H_5_)_x_ + xHCl), preventing unexpected reactions with water or moisture (VCl_3_ + xH_2_O → VCl_3−x_(OH)_x_ + xHCl). According to the electrochemical series, the reduction potential of V^4+^ → V^3+^ is approximately 0.337 V, which is lower than that of [Fe(CN_6_)]^3−^ → [Fe(CN_6_)]^4−^ (0.358 V) [33]. Therefore, V^3+^ can be oxidized to V^4+^, while [Fe(CN_6_)]^3−^ can be reduced to [Fe(CN_6_)]^4−^. The reaction was completed after 1 h under a heating supplement at 80 °C, and the resultant was a green precipitate of VFCN (Prussian green).

**Figure 1:**
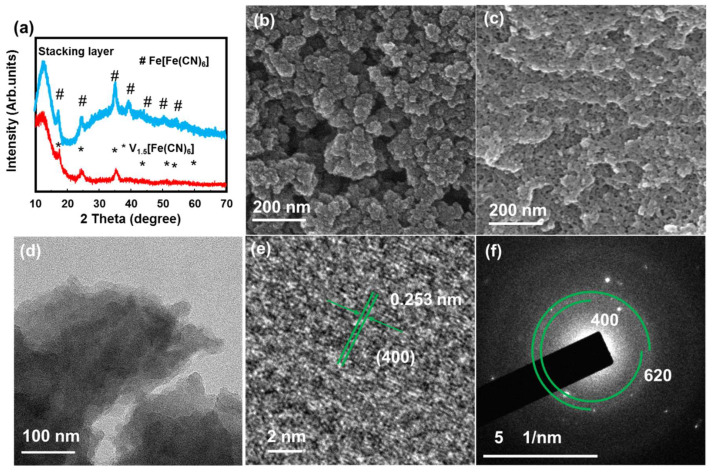
(**a**) X-ray diffraction (XRD) patterns of VFCN and FFCN; (**b**) Scanning electron microscopy (SEM) image of VFCN; (**c**) SEM image of FFCN; (**d**) Transmission electron microscopy (TEM) image; (**e**) high resolution TEM image and (**f**) selected area electron diffraction patterns of VFCN material.

Figure 1a shows the XRD pattern of the VFCN and Fe[Fe(CN)_6_] (FFCN) samples, which matches the V_1.5_[Fe(CN)_6_] pattern with a cubic structure (JCPDS#00-042-1440). In addition, a peak at ~12° is observed, which can be the (110) plane in VFCN or the spacing distance of the stacking layer observed in the synthesis of several 2D materials [34,35,36]. The FeFe(CN)_6_ was also confirmed with peaks corresponding to JCPDS #01-0239 [37]. It should be noted that the XRD patterns of these two materials were quite similar because of the same cubic structure, with a small difference in the lattice constant around 10.2 Å [38]. The SEM images in Figure 1b,c show aggregated VFCN and FFCN with a size distribution below 100 nm. To further reveal the morphology of the VFCN sample, TEM images were obtained, as shown in Figure 1d–f. As shown in the TEM images in Figure 1d, the obtained sample had a nanosheet structure with a size of a few tens of nanometers. The lattice spacing of the (400) plane was well measured in Figure 1e in addition to the amorphous points on the surface of the layer, which could be due to the small oxidation of the surface. The SAED pattern in Figure 1f also supports the low crystallinity of the as-prepared VFCN owing to the small particle size and surface oxidation of the sample. It can be considered that the prepared VFCN sample is a nanosheet with a cubic Prussian blue structure and an amorphous surface. Based on the aforementioned information, VFCN and FFCN have a similar cubic structure with a lattice constant of ~10.2 Å. However, VFCN revealed a less crystalline structure than FFCN.

**Figure 2:**
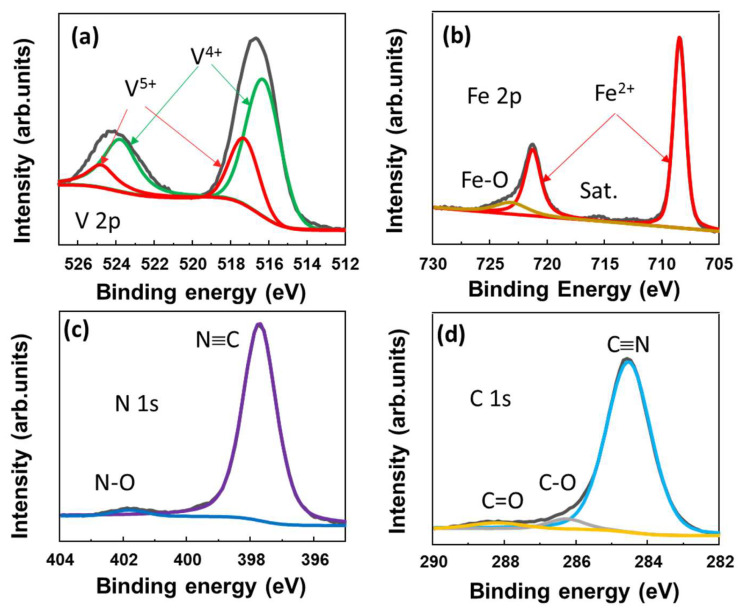
X-ray spectroscopy spectra of VFCN with elemental scans of (**a**) V 2p; (**b**) Fe 2p; (**c**) N 1s; and (**d**) C 1s core levels.

To further investigate the surface of the VFCN material, the X-ray photoelectron spectroscopy (XPS) spectra are shown in Figure 2a–d. The core levels of V, Fe, N, and C were well recorded at ~516.3, 708.4, and 398.7/284.5 eV, which indicate the V^4+^, Fe^2+^, and N≡C binding states, respectively. The Fe^2+^ and V^4+^ states can be described as V^3+^ being oxidized to V^4+^ and Fe^3+^ in [Fe(CN)_6_]^3−^ being reduced to Fe^2+^, resulting in [Fe(CN)_6_]^4−^. In addition, the small peaks of V^5+^ were deconvoluted at ~517.4 and 524.8 eV, indicating the oxidation of V on the surface, as illustrated in Figure 2a. This is consistent with the small Fe^3+^, N-O, and C-O/C=O peaks, both of which indicate that the surface of the material was slightly oxidized. Therefore, it can be concluded that VFCN Prussian green was well prepared using the redox reactions of VCl_3_ and K_3_Fe(CN)_6_ (KFCN) in the solvothermal process.

**Figure 3:**
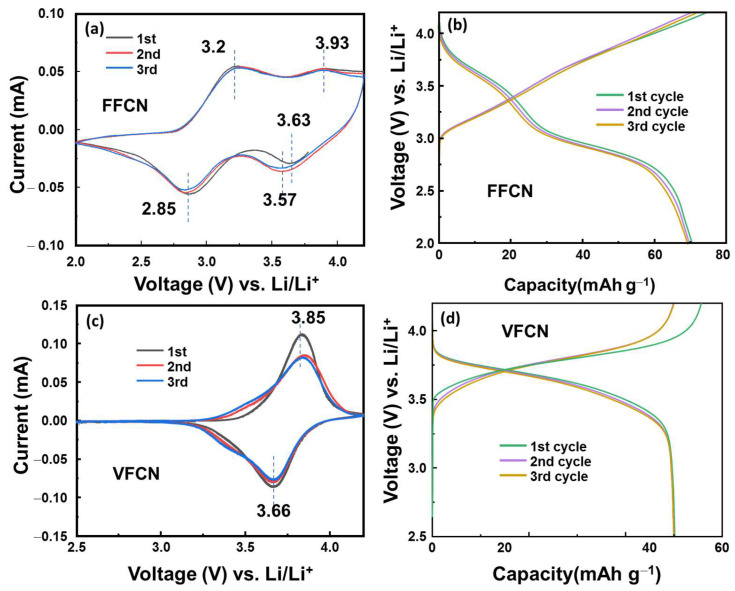
(**a**) Cyclic voltammetry (CV) curve measured at 1 mV s^−1^ and (**b**) initial voltage profiles at 50 mA g^−1^ of FFCN cathode. (**c**) CV curve and (**d**) initial voltage profiles of VFCN cathode.

Half-cell-type lithium-ion batteries were utilized to evaluate the electrochemical properties of VFCN, which were compared to those of the FFCN cathode material. Figure 3a–c show their CV curves in the range of 2.5–4.2 V vs. Li/Li^+^ at 0.1 mV s^−1^. FFCN as a typical PBA showed four redox peaks, which are related to the redox reactions of low spin and high spin Fe^2+^/Fe^3+^ at ~3.6/3.9 and 2.85/3.2 V vs. Li/Li^+^ [34,39,40]. However, VFCN shows only two stable redox potential peaks, which were recorded at 3.66 and 3.85 V vs. Li/Li^+^. This implies that the high-spin state of Fe^2+^/Fe^3+^ was significantly reduced or inactivated. Furthermore, Pan et al. reported that only Fe was active and V was inactive in the electrochemical process [32]. The small change from the first cycle could be due to a side reaction related to the interaction of Li ions on the VFCN amorphous surface and formation of a solid electrolyte layer in the first cycle [41,42]. Figure 3b,d show the plots of the voltage profiles of FFCN and VFCN at a current of 50 mA g^−1^, respectively. The FFCN electrode shows two voltage flatforms at ~2.8 and ~3.5 V during the discharge process, which could be related to the two redox states of high and low spin Fe^3+^ ions. Therefore, the voltage gap was mostly distributed between ~2.6 and 3.8 V. In contrast, the VFCN electrode shows a stable voltage flatform from ~3.2 to 3.8 V due to the inactive high-spin Fe^2+^ ions during the electrochemical reactions. Thus, the VFCN cathode has higher redox potential than that of the FFCN cathode. Therefore, it can be concluded that the presence of V and deactivated high-spin Fe^2+^ ions stabilized the electrochemical reactions, resulting in the formation of a stable voltage flatform.

**Figure 4:**
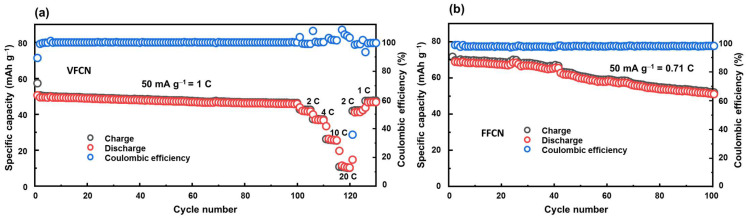
(**a**) Cycling test for 100 cycles at 50 mA g^−1^ (corresponding to 1 C) and rate performance at 2, 4, 10, and 20 C of VFCN cathode, and (**b**) cycling test for 100 cycles of FFCN cathode at 50 mA g^−1^ (corresponding to 0.71 C).

The cycling stabilities of the VFCN and FFCN cathodes were determined via cycling tests, as illustrated in Figure 4a,b, respectively. The VFCN cathode was run at 50 mA g^−1^ (corresponding to 1 C) for 100 cycles, and then high-current rate performance tests at 2, 4, 10, and 20 C were performed. This shows that the VFCN cathode possesses high stability during cycling, and the capacity is maintained at ~90.7% of the initial discharge capacity after 100 cycles. The high stability of VFCN cathode could be due to the more stable VFCN structure resulting from the existence of the V^4+^ ions and the V-O binding. As discussed above, the V ions are not involved in the electrochemical process. Therefore, they can act as stable elements in the VFCN lattice structure. This helps the restoration of the material during lithium insertion/extraction, leading to high stability during electrochemical process. The capacities decreased to 82.4, 73.0, 52.5, and 22.1% at 2, 4, 10, and 20 C, respectively. The capacities were highly responsive to changes in the current rate. Hence, after the high-current-rate test, the VFCN cathode showed a fast restoration from 20 to 2 and 1 C, where the capacity retention changed from low capacity values of ~15 mAh g^−1^, and then it was immediately restored to a stable capacity. Interestingly, at 1 C, the capacity was restored to 47.3 mAh g^−1^, which is 92.7% of the initial discharge capacity and is similar to the capacity at the 100th cycle (90.7%). This result indicates that the decrease in capacity may not be related to the degradation of the cathode material, but to the degradation of the lithium foil or electrolyte [43,44]. In contrast, the FFCN cathode showed a higher capacity of ~72 mAh g^−1^ in the first cycle, and then the capacity decreased to 72% compared to the first capacity value at the 100th cycle. It is noted that VFCN has a lower capacity than FFCN; however, the VFCN electrode demonstrated high stability and a high-voltage flatform, which is applicable to LIB cathodes.

**Figure 5:**
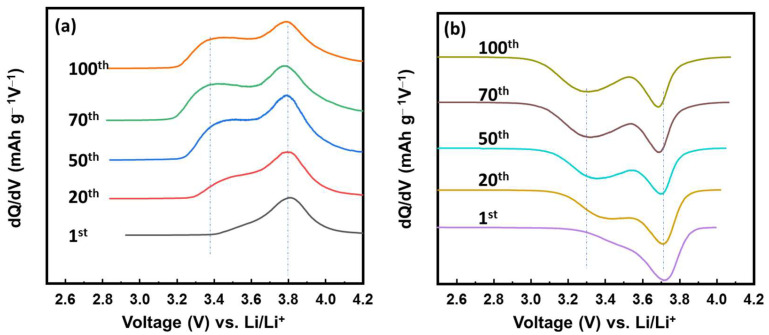
Differential capacity plots of VFCN cathode for the (**a**) charge and (**b**) discharge process during cycling.

To investigate the changes in the electrochemical process of the VFCN cathode, differential capacity plots (DCP) for the cathodic and anodic processes are shown at the 1st, 20th, 50th, 70th and 100th cycles in Figure 5a,b. In the charging process, the oxidation peak started changing from the 20th cycle and was maintained from the 50th cycle. The oxidation peak was divided from a single peak at ~3.8 V vs. Li/Li^+^ to two peaks at ~3.4 and 3.8 V vs. Li/Li^+^. Consistent with this change, the discharge process shown in Figure 5b shows a similar tendency. The single reduction peak at ~3.7 V vs. Li/Li^+^ was divided into two reduction peaks at ~3.3 and 3.7 V vs. Li/Li^+^. The couple peaks at 3.3/3.4 and 3.7/3.8 V are related to the redox potential of high spin and low spin Fe^2+^ ions, respectively [34,39,40]. Thus, the lithium ion insertion/desertion changed the states of Fe from only low-spin to the mixed states of high- and low-spin Fe ions. This could be due to the effect of lithium ions, which partially transferred their dynamic momentum to the VFCN structure. Lithium or some alkaline metal ions (Na, K) have been reported as effective ions for the phase modification of transition metal chalcogenides from 2H to 1T [45]. In our study, the Li ions affected the spin of Fe, in which the balance of low and high spins was established after 50 cycles. It should be noted that the capacity did not change because of this phenomenon, as shown in Figure 4. Therefore, the electrochemical properties of VFCN changed from low-spin to mixed low- and high-spin states of Fe ions after 50 cycles.

**Figure 6:**
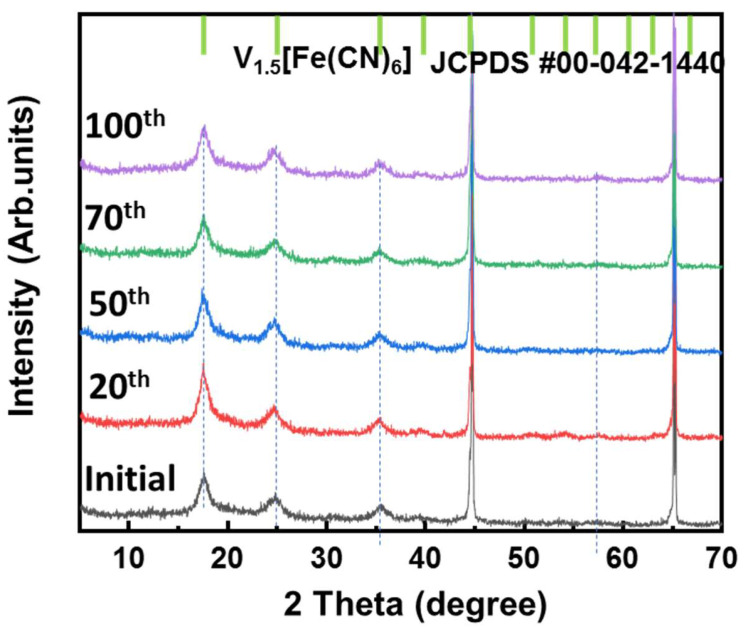
Ex situ XRD patterns of VFCN cathode at initial state and after cycling for 20, 50, 70, and 100 cycles.

Ex situ XRD measurements were conducted to investigate the change in the VFCN structure during different cycles from the initial to 100th cycle, as illustrated in Figure 6. All samples showed only the structure of VFCN with the same peak positions corresponding to JCFPD #00-042-1440 of V_1.5_[Fe(CN)_6_]. The DCP results indicate that the electrochemical properties changed after 50 cycles. However, this change was not observed in the structure of VFCN. This result suggests that Li-ion insertion and desertion only changed the spin of Fe ions, but did not affect the lattice structure of VFCN. Therefore, no changes were observed in the XRD pattern. Finally, the VFCN structure was stable during the insertion/extraction of Li ions.

**Figure 7:**
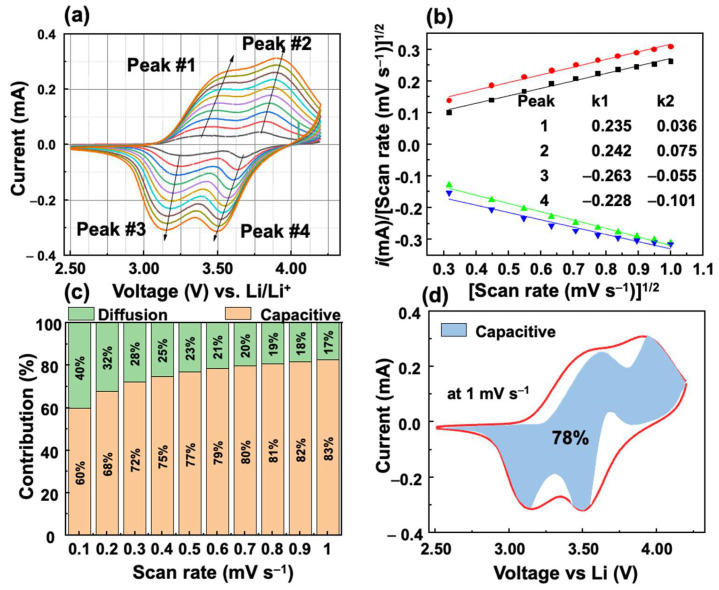
Kinetic analysis of Li storage behavior of VFCN cathode: (**a**) CV curves at different scan-rate voltages from 0.1 to 1.0 mV s^−1^. (**b**) Fitted lines of $\frac{i}{v^{1/2}}$ vs. $v^{1/2}$ at different scan-rate voltages with the calculated k_1_ and k_2_ values (inset). (**c**) Contribution ratio of capacitive and diffusion-controlled process of peak#3. (**d**) Capacitance contribution at 1.0 mV s^−1^.

The kinetic analysis of the VFCN cathode was performed using Dunn’s method, as shown in Figure 7. Four points from 1 to 4 were selected as the main peaks from the oxidation and reduction processes, as shown in Figure 7a. According to the relation between current *i* and voltage scan rates (*v*),

(1) $$i=k_{1}v+k_{2}v^{1/2}$$

where *k*_1_, *k*_2_ are constants representing the capacitive and diffusion contributions to the current, respectively. By dividing Equation (1) by the square root of *v*:

(2) $$\frac{i}{v^{1/2}}=k_{1}v^{1/2}+k_{2}$$

The factors *k*_1_ and *k*_2_ were calculated from the slope and intercept of the fitted lines of $\frac{i}{v^{1/2}}$ and $v^{1/2}$, as shown in Figure 7b. *k*_1_ is always larger than *k*_2_, which indicates a major contribution of the capacitive behavior. The contributions of the diffusion- and capacitive-controlled processes of the VFCN cathode at peak#3 were calculated at different scan rates from 0.1 to 1 mV s^−1^, as shown in Figure 7c. The capacitive behavior is observed to be a major contributor to the current that increased from 60 to 83% when the scan rate is increased. The significant contribution of the capacitive behavior-controlled process indicates that the high performance of the VFCN cathode originates from the capacitive properties of the metal–organic framework between V, Fe, and CN. To provide an overview of the contribution of the capacitive process over the entire cycle, 100 points for each anodic and cathodic scan were extracted to calculate *k*_1_ and *k*_2_, and the capacitive contribution to the current. Figure 7d shows the capacitive behavior contribution of 78% for the whole cell performance at 1 mV s^−1^. Therefore, the VFCN cathode exhibited the main pseudo-capacitance behavior for Li-ion storage.

Table 1 summarizes the electrochemical performance of Prussian blue and some metal–organic framework structures including V- and Fe-based metals as cathode materials in LIBs. There are rare reports on the Prussian blue in LIB due to its low performance and low redox potential. FFCN or some Fe-based Prussian blues typically have two redox potentials at ~2.8/3.1 and 3.4–3.8/3.9 V. Furthermore, the performance of these cathodes was obtained at a low current rate range of ~10–25 mA g^−1^. As shown in Table 1, the organic-based cathodes showed a stability at lower current rates than oxide-based cathodes including V_2_O_5_. In this work, however, the redox potential is much improved with the incorporation of V ions, which also exhibited stable cyclability. With the achievements in stability and redox potential, VFCNs can have a significant impact on lithium storage applications.

## 3. Materials and Methods

### 3.1. Chemicals

Iron (III) chloride (FeCl_3_, anhydrous), vanadium (III) chloride (VCl_3_), potassium ferrocyanides (KFCN, K_3_Fe(CN)_6_.4H_2_O), and 1-methyl-2-pyrrolidone (NMP, anhydrous) and polyvinylidene fluoride (PVDF, MW 534,000) were purchased from Sigma-Aldrich Inc. (St. Louis, MO, USA). Super-P amorphous carbon black (C, ~40 nm), ethylene carbonate (EC), propylene carbonate (PC), and absolute ethanol were purchased from Alpha Aesar, Inc. (Haverhill, MA, USA).

### 3.2. Vanadium Ferrocyanides (VFCN) Synthesis

VFCN was prepared using the solvothermal method. KFCN (0.73 g) was dissolved in 100 mL of deionized (DI) water at 80 °C under continuous stirring. VCl_3_ (0.16 g) was dissolved in 20 mL of absolute ethanol and slowly added to the above solution. The solution appeared yellow-green and then changed to green. The reaction was completed after 1 h. The precipitate was then washed with DI water (×2) and ethanol (×4) via centrifugation. The VFCN powder was obtained after drying for 2 d in a freeze dryer (Labconco Corp., Kansas, MO, USA).

### 3.3. Ferric Ferrocyanides (FFCN) Synthesis

To compare with traditional PBAs, FeFe(CN)_6_ was prepared using a process similar to that used for VFCN, the VCl_3_ being replaced by FeCl_3_ (same mole ratio). The resulting precipitate was FeFe(CN)_6_, which is blue in color and named FFCN.

### 3.4. Materials Characterizations

The morphologies and sizes of the materials were analyzed by scanning electron microscopy (SEM; Hitachi S4700, Tokyo, Japan) at an accelerating voltage of 5 kV and transmission electron microscopy (TEM; TECNAI G2F30, FEI Corp., Hillsboro, OR, USA) at an accelerating voltage of 200 kV. High-resolution X-ray diffraction (XRD; SmartLab, Rigaku, Tokyo, Japan) was applied to investigate the structure of the material. The XRD patterns were recorded over a 2θ range of 10–70°. X-ray photoelectron spectroscopy (XPS; Kratos Analytical Ltd., Manchester, UK) was used to measure the composition and elemental core level.

### 3.5. Electrochemical Measurements

The cathode materials were assembled in a half-cell structure with a lithium metal anode using coin-type cells (CR 2032, Rotech Inc., Gwangju, Republic of Korea). Cathodes were prepared using a doctor blade on aluminum foil using a slurry of 80% active materials with 10% conductive Super P carbon and 10% PVDF as a binder. Then, the electrodes were dried overnight at 70 °C under vacuum conditions and punched into 12 mm diameter circular disks. The loading of the active materials was ~1.5–2.0 mg cm^−2^. Polyethylene membranes were employed as separators, and a solution of 1M LiPF_6_ in a mixed solvent (EC:DEC, 1:1 by volume) was used as the electrolyte. The galvanostatic electrochemical charge/discharge performance of the LIB cells was evaluated using a battery cycle tester (WBCS3000, WonAtech, Seocho-gu, Seoul, Republic of Korea) over the voltage range of 2.5–4.2 V versus Li/Li^+^. Cyclic voltammetry (CV), across a voltage range of 2.5–4.2 V, was performed using ZIVE MP1 (WonAtech, Seocho-gu, Seoul, Republic of Korea). The differential analysis of charge and discharge after 1, 20, 50, 70, and 100 cycles was extracted from the cycling test using IVMAN differential analysis software. All the specific capacities were calculated based on the weights of the active materials.

## 4. Conclusions

In this study, vanadium ferrocyanide nanosheets were successfully synthesized as a Prussian green analog material. The synthesis was achieved via the redox reaction of VCl_3_ with KFCN under a solvothermal process. The resulting VFCN was investigated as a cathode material in LIBs, and it demonstrated high stability, high redox potential, and stable voltage flatform. The VFCN cathode exhibited a reversible capacity of ~50 mAh g^−1^ and ~100% restoration in capacity, even after high-rate performance. The ex situ differential capacity plots revealed that the spin of Fe ions changed from low spin to mixed states of low and high spins, which could be because of the effectiveness of Li ions during insertion/desertion. The ex situ XRD patterns confirmed the high stability of the VFCN structure. In addition, kinetic analysis showed that the capacitance behavior was the major mechanism for Li storage of the VFCN electrode. The simplicity of the process, low cost of material, high stability, and high-voltage flatform of VFCN can contribute to future economic and environmentally friendly LIBs.

## Figures and Tables

**Table 1:** Comparison of VFCN with some typical Prussian blue and metal–organic frameworks including V- and Fe-based metals.

Materials	Redox Potential (V) vs. Li/Li^+^	Specific Capacity (mAh g^−1^)/Rate (mA g^−1^)	At Cycle Number	Reference
Fe^(2+)^Fe^(2+)^(CN)_6_	2.8/3.1 and 3.8/3.9	96/25	50	[46]
Fe^(3+)^_4_[Fe^(2+)^(CN)_6_]_3_	2.7/3.1 and 3.4/3.9	71/25	50	[46]
V_2_O_5_	2.3/2.5 and 3.4/3.7	169/133	50	[47]
MIL-101(Fe)	2.6/3.2	72/15	100	[48]
MIL-53(Fe)	~2.7/3.0	70/8	50	[49]
Na_1.1_(VO)_1.07_Cu_0.35_Fe(CN)_6_	~2.5/2.7 and 3.4/3.5	93/50	50	[50]
FFCN (Fe^(3+)^Fe^(3+)^(CN)_6_)	2.8/3.2 and 3.6/3.9	52/50	100	This work
VFCN	3.66/3.85 3.3/3.4 and 3.7/3.8 (After 50 cycles)	48/50	130	

## Data Availability

The data presented in this study are available on request from the corresponding author.

## Sample Availability

Samples of the compounds includingVFCN are available from the authors.
